# The modified dental anxiety scale: UK general public population norms in 2008 with further psychometrics and effects of age

**DOI:** 10.1186/1472-6831-9-20

**Published:** 2009-08-26

**Authors:** Gerry M Humphris, Tom A Dyer, Peter G Robinson

**Affiliations:** 1 Health Psychology, Bute Medical School, University of St Andrews, UK; 2 Department of Oral Health and Development, University of Sheffield, Sheffield, UK

## Abstract

**Background:**

The Modified Dental Anxiety Scale (MDAS) is a brief, self-complete questionnaire consisting of five questions and summed together to produce a total score ranging from 5 to 25. It has reasonable psychometric properties, low instrumental effects and can be integrated into everyday dental practice as a clinical aid and screen for dental anxiety. The objectives were to (i) produce confirmatory evidence of reliability and validity for the MDAS, (ii) provide up-to-date UK representative norms for the general public to enable clinicians to compare their patients' scores, (iii) to determine the nature of the relationship between dental anxiety and age.

**Methods:**

Telephone survey of a representative quota sample of 1000 UK adults (>18 years of age) conducted between 7–21 April, 2008.

**Results:**

Attrition of potential participants was high in the recruitment process, although bias was minimal. Estimated proportion of participants with high dental anxiety (cut-off score = 19) was 11.6%. Dental anxiety was four times greater in the youngest age group (18–39 yrs) compared to older participants (60+ yrs), controlling for sex, social class and self-reported dental visiting behaviour confirming previous developed-world reports.

**Conclusion:**

The scale's psychometrics is supportive for the routine assessment of patient dental anxiety to compare against a number of major demographic groups categorised by age and sex. Dental anxiety was high in younger compared to older people.

## Background

Dental anxiety remains a barrier to dental care for a consistent proportion of the population [1]. This is disappointing as improvements in oral health over the past three decades have reduced restorative treatment in many developed countries. A reduction in dental anxiety levels had been expected as anxiety is related to experience of invasive treatment procedures. As very often in behavioural science the simple explanation is insufficient to explain complex human psychological processes.

One aid to explaining, identifying and reducing dental anxiety is a good measure of the condition that can be used in clinical and research settings. Clinicians need to diagnose the condition and evaluate strategies to reduce it. In interpreting trends and making comparisons, researchers need to attend carefully to measurement issues.

Several methods of assessment have been used. For example, the UK Adult Dental Health Survey contained a question about nervousness about visiting the dentist [2]. The reports are difficult to interpret as the measurement properties of this item have not been investigated. Conversely, measures based on the Dental Fear Survey [3] consist of many questions and are more suitable for intensive research purposes than routine clinical use [4]. Other measures are based on Corah's Dental Anxiety Scale (CDAS) [2]. The CDAS unfortunately does not enquire about local anaesthetic injection, which is a focus for some patients' anxiety [5].

Psychometric details for another dental anxiety measure, the Modified Dental Anxiety Scale (MDAS) are available for the UK [6]. This measure, modelled on the original CDAS, includes a question on local anaesthesia. The original data for this modified scale were published in 1995 and some improvements in oral health may have occurred. Hence a possible outcome is less invasive dental treatment (e.g. regional improvement in Northeast England) [7]. In addition, the original norms included a variety of samples including participants from student groups, various dental services including general and industrial dental practice. No large sample was presented from the general public. A new survey was considered appropriate to update the norms and provide a more comprehensive picture for a large sample of the general public as this would be instructive for researchers and clinicians. An additional question to address was whether younger people have lower anxiety levels than their older counterparts. The original set of norms in the UK demonstrated very similar anxiety levels in the first 4 decades of adult life. Some reports have indicated that younger adults may show lower dental anxiety than their more middle aged counterparts [6,8]. Such an effect, if repeated in the UK public, might suggest that improved oral health and fewer treatment interventions may be reflected in the UK public's perception of dental care.

The Modified Dental Anxiety Scale is a brief, 5 item questionnaire with a consistent answering scheme for each item ranging from 'not anxious' to 'extremely anxious' [6]. It is summed together to construct a Likert scale with a minimum score of 5 and a maximum of 25. It is the most frequently used dental anxiety questionnaire in the UK [9] and does not increase patient fears when completed [10,11]. Existing data suggest that completion of the questionnaire can significantly reduce state anxiety in the practice setting [12]. It has good psychometric properties, is relatively quick to complete and scoring is easy [13,14]. A cut-off value of 19 and above has been determined empirically [6] to indicate high dental anxiety that may require special attention by dental personnel. The measure has been used in research studies and helped to contribute to our knowledge of this important dental related psychological construct. It is one of a number of instruments that have been designed to help study the properties of this unpleasant feeling [4]. As previously stated, the scale is based on the original Corah's Dental Anxiety Scale (CDAS) [15] for which conversion tables have been published to compare values between the two instruments [16] The MDAS has been translated into a number of world languages, many of which have published psychometrics (Spanish [17], Turkish [18], Greek [19], Chinese [20]).

A number of reports have presented data of UK samples against which clinicians may compare the scores of their patients [11-13]. However, as these samples may not have been representative, new data representing UK norms would provide a valuable comparator for patient assessments. Further, researchers or clinicians may benefit from access to percentiles tabulated across major demographic groups to enhance comparison. The advantage of percentiles is that they can identify the rarity of a patient's score, and hence provide information supplementary to being above or below a cut-off [21]. Hence the aims of this paper were threefold: first, to confirm the factorial validity of the MDAS and present a precise reliability estimate; second, to report a set of norms for the public within the UK for clinicians to utilise for comparison with their patients' scores; and lastly, to determine the nature of the relationship between dental anxiety and age.

## Methods

### Design

A telephone survey was undertaken between 7–21 April 2008 by a market research company (GfkNOP) using a structured interview of a representative quota sample (n = 1000) of UK adults (18 years and over).

### Sampling

Potential participants were telephoned using random dialling in postcodes to obtain a sample that was representative of the Office for National Statistics mid 2005 population estimates for the 4 individual countries in the UK. To achieve a quota sample of 1000 UK adults, 6937 unique telephone numbers were called. Of the numbers called, 1704 were called back as they were either busy or engaged (n = 466) or the participant requested the interview take place at a different time (n = 1238). Only 91 calls resulted in no contact being made (due to wrong numbers, no answer after a number of call attempts, or the number being out of service). Of those contacted, 5828 declined to participate and a further 18 stopped the interview providing a response rate of 14%.

### Measures

The MDAS asks participants to rate their emotional reaction to the prospect of a dental visit the day previous, then when in the waiting room, receipt of drilling, scaling and a local anaesthetic injection. Precoded responses range from 'not anxious' (scoring 1) to 'extremely anxious' (scoring 5). Reliability of the English language version of the MDAS is good (internal consistency = 0.89; test-retest = 0.82). The scale can be downloaded: .

### Procedure

Potential participants were telephoned out of normal working hours. The subject matter, purpose and the likely duration of the survey were explained. Potential participants were informed that they could decline involvement in the survey at any stage during or after the interview. Having been asked about demographic data, participants were asked the 5 questions of the MDAS. In addition, other questions were asked on the use of dental therapists, the findings of which will be reported elsewhere.

### Statistical analysis

Data were analysed using SPSS v16, and reliability analysis conducted with FACTOR [22]. The scale was factor analysed (principal factor method) and Horn's parallel analysis was run to determine the factorial structure [23]. Confirmatory analyses were also completed using AMOS17 [24]. Means and standard deviations were calculated across the major demographic factors and self-reported visiting. A set of percentiles was prepared across gender and major age groups. A threshold of 19 and above was adopted, as the level for which is it likely that a dental practitioner would consider using additional approaches to manage the patient such as relaxation, systematic desensitisation or pharmacological adjunct. Item frequencies were inspected for male and female samples and the ratings examined across samples to determine if individuals differ by anticipatory (i.e. contemplating a visit to the dentist the next day and sitting in the waiting room) and treatment related items using the Wilcoxon Ranks Sign test. The proportion of individuals who scored 19 and above was calculated across the demographic and behavioural variables. Cross tabulations were performed with categorical variables. Multiple logistic regression was employed to establish the independent association of demographic factors (gender, age, educational level, social class and self-reported dental visiting) on the dichotomous classification of high (≥19) and moderate to low dental anxiety (≤18). Significance level was set at the conventional 5%.

Ethical approval for the study was granted by the University of Sheffield, UK.

## Results

The survey completion was excellent with no missing values for the MDAS questionnaire. More than 99% of participants supplied their age and social class. One person declined to estimate past dental visiting behaviour and 18 did not provide information about their educational background (see Table 1). The survey sampling was successful in retrieving participants from all adult age groups and genders. 491 or 51% of the sample were female reflecting closely the UK proportion. Similarly, age groups 18 to 34 years, 35 to 54 years and 55 years plus matched UK proportions of 32%, 34% and 34% respectively. Somewhat more non-manual participants were collected, 59% compared with the UK percentage of 49%.

**Table 1:** Frequency breakdown and N size for participant sample including MDAS means (SD) and percent ≥ 19

	**N**	**%**	**Mean**	**SD**	**% ≥19**
		
**Total**	963	100	10.39	5.46	11.6
					
**Sex**					
Male	472	49.0	9.22	4.94	8.3
Female	491	51.0	11.52	5.69	14.9
					
**Age (years)**					
18–29	189	19.6	11.62	5.44	14.3
30–39	179	18.6	11.61	5.88	17.3
40–49	170	17.7	10.28	5.34	12.4
50–59	175	18.2	10.29	5.33	10.3
60–69	119	12.4	10.03	5.27	8.4
70+	125	13.0	7.64	4.29	4.0
Refused	6	0.6	6.33	1.51	0.0
					
**Visiting the dentist**					
Regular	686	71.2	9.94	5.12	9.3
Occasional check up	134	13.9	10.75	5.38	12.7
When in pain/or trouble	90	9.3	12.39	6.59	22.2
Never been	52	5.4	12.17	6.73	21.2
Refused	1	0.1			
					
**Education**					
No school	30	3.1	10.37	5.92	16.7
Secondary	97	10.1	10.45	5.37	10.3
Secondary with qualifications	239	24.8	10.54	5.84	14.2
College A levels	185	19.2	10.10	5.41	11.4
Technical GNVQ	84	8.7	10.32	5.74	15.5
University degree	151	15.7	10.28	5.01	7.9
University post grad degree	158	16.4	10.42	5.10	8.2
DK/Refused	19	1.9	12.16	6.41	21
					
**Social class**					
A	44	4.6	10.48	6.41	15.9
B	180	18.7	10.14	5.1	7.8
C1	342	35.5	10.15	5.07	9.1
C2	159	16.5	11.14	5.59	17.0
D	82	8.5	11.91	5.88	17.1
E	148	15.4	9.76	5.94	12.8
Refused	8	0.8	7.38	3.02	0

To satisfy the first study objective we confirmed the psychometric properties of the MDAS. An exploratory factor analysis using parallel bootstrapping to derive simulated eigenvalues from random samples for comparing with the observed data was conducted. The Kaiser-Meyer-Olkin test (indicates if sufficient common variance exists to merit factor analysis) gave a satisfactory high value of 0.842. The factor analysis demonstrated a clear unity factor structure (eigenvalue for first factor = 3.69, which is equivalent to three times the average amount of variance contained within this factor as the rest of the covariance matrix, and eigenvalue for second factor = 0.51) demonstrating that the scale can be considered uni-dimensional for practical purposes. Random eigenvalues derived from the bootstrapping procedure showed that 2 factors would have been selected (eigenvalues of 1.13 and 1.06) if the popular unity criteria for setting the number of factors had been adopted. The single factor contained 93% of the explained variance. This result was supported partially by testing the model constrained to a single latent factor. Fit statistics showed excellent correspondence between the model and raw data with just 3 error covariances relaxed (chi square = 3.89; df = 2; *p *= .14; CFI = .999, TLI = .997, RMSEA = .031). The internal consistency coefficient of the scale was excellent (0.957, 95%CI 0.953, 0.961).

The proportion of participants at or above the threshold of 19 on the MDAS showed considerable range across many of the demographic variables and past dental visiting patterns. This variation is explored in more detail in the multiple logistic regression analysis below. The cross-tabulation of educational level appeared not to be associated with the categorisation of those into the two dental anxiety groups (high *versus *moderate or low). However, this was somewhat misleading as there were 7 categories of educational level which were not ranked into a clear order. For example the 'technical GNVQ' qualification was regarded as a vocational award as opposed to achieving a high academic standard. For the purposes of the multiple logistic regression analysis this factor was dichotomised into those participants with a university education as opposed to those without. Similarly the social classification variable was split into manual and non-manual categories.

The individual item frequencies (see Table 2) showed that the majority of men were 'not anxious' on anticipatory events (visiting the dentist tomorrow and sitting in waiting room) and scale and polish compared with receiving the drill and local anaesthetic injection. The majority of women however were at least 'slightly anxious' with all items in the questionnaire (with exception of 'scale and polish'). The replies of each sex to each item was tested against the other items in the scale by the Wilcoxon Ranks Sum Test. All items were rated significantly different from each other (*p *< .001) with the exception of the two anticipatory items (Items 1 and 2) which displayed similar ratings for both men (*p *= .056) and women (*p *= .272).

**Table 2:** Item frequency breakdown of MDAS across male and female samples

*MALE*										
Question	Visit Tomorrow		Waiting Room		Use of Drill		Scale and Polish		Injection	
	N	%	N	%	N	%	N	%	N	%
not anxious	**283**	**60**	**270**	**57**	**188**	**40**	**346**	**73**	**212**	**45**
slightly anxious	103	22	108	23	132	28	79	17	136	29
fairly anxious	**31**	**7**	**41**	**9**	**59**	**13**	**19**	**4**	**56**	**12**
very anxious	32	7	29	6	52	11	13	3	40	8
extremely anxious	**23**	**5**	**24**	**5**	**41**	**9**	**15**	**3**	**28**	**6**
										
Base N (%)	472	100	472	100	472	100	472	100	472	100
										
*FEMALE*										
Question	Visit Tomorrow		Waiting Room		Use of Drill		Scale and Polish		Injection	
	N	%	N	%	N	%	N	%	N	%

not anxious	**217**	**44**	**209**	**43**	**120**	**24**	**307**	**63**	**135**	**27**
slightly anxious	108	22	122	25	124	25	88	18	135	27
fairly anxious	**71**	**14**	**51**	**10**	**63**	**13**	**49**	**10**	**86**	**18**
very anxious	45	9	60	12	94	19	24	5	71	14
extremely anxious	**50**	**10**	**49**	**10**	**90**	**18**	**23**	**5**	**64**	**13**
										
Base N (%)	491	100	491	100	491	100	491	100	491	100

The second objective of the study was completed by calculation of percentile norms for MDAS scores for the UK population by age and gender (Table 3). To aid interpretation of the table the values of the MDAS at 19 and above have been formatted in bold type at the appropriate percentile points. Three broad age groups are presented. The precision of the location of the cut-off value against the percentile scale was considered to be important. Such precision would have been compromised had all 6 age groups been listed. From inspection of the cut-offs at each age group across the sexes there appeared to be an interaction between age group and gender. Almost 15% of women scored above the cut-off for the MDAS whereas only 8% of men scored this highly. On further inspection of the tables and single items it was clear that approximately twice the number of women (18 and 13%, respectively) compared with men (9 and 6%, respectively) were extremely anxious about drilling and local anaesthetic injection. Similarly, 10% of women stated that they were extremely anxious about anticipating a dental visit the next day and sitting in the waiting room compared with 5% of men.

**Table 3:** Means, medians (SDs) and percentiles for total MDAS scores broken down by sex and age group

	M 18–39	M 40–59	M 60+	F 18–39	F 40–59	F 60+	
N	161	185	122	207	160	122	
Mean	10.84	8.99	7.52	12.21	11.79	10.09	
Median	9	8	6	12	11	9	
SD	5.63	4.57	3.81	5.61	5.74	5.57	
Percentiles							Percentiles
5	5	5	5	5	5	5	5
10	5	5	5	5	5	5	10
15	5	5	5	6	5	5	15
20	6	5	5	6	6	5	20
25	6	5	5	7	7	5	25
30	7	6	5	8	7	6	30
35	7	6	5	9	8	6	35
40	8	7	5	9	9	7	40
45	9	7	5	11	10	8	45
50	9	8	6	12	11	9	50
55	10	8	6	13	11	9	55
60	11	9	7	14	12	9	60
65	12	9	7	14	13	10	65
70	13	10	8	16	15	11	70
75	14	11	9	17	16	14	75
80	16	11	10	17	17	15	80
85	18	13	12	**19**	**19**	16	85
90	**19**	17	13	**20**	**21**	**19**	90
91	**20**	18	13	**21**	**22**	**19**	91
92	**21**	18	13	**21**	**22**	**20**	92
93	**22**	**19**	14	**21**	**22**	**21**	93
94	**22**	**19**	16	**22**	**22**	**22**	94
95	**23**	**20**	16	**22**	**22**	**24**	95
96	**23**	**20**	17	**23**	**23**	**25**	96
97	**23**	**20**	**19**	**23**	**24**	**25**	97
98	**25**	**21**	**21**	**24**	**25**	**25**	98
99	**25**	**24**	**23**	**25**	**25**	**25**	99

Note: bold type indicates all values ≥ 19

The final objective of the study was achieved from the results of the multiple logistic regression analysis (see Table 4) and confirmed that high dental anxiety was more common among younger adults independent of sex, occupation and education. The latter three factors were relatively of less explanatory value than age of respondent. Age was the strongest predictor of those with high anxiety (≥ 19 score) with participants aged 18–39 over four times more likely to be dentally anxious than those 60 years or older. Middle aged participants (40–59 years) were 3 times more likely to score at or over the cut-off.

**Table 4:** Logistic regression to predict those at cut-off of 19 or above on the MDAS with variables: age group, sex, social class, education and self reported visiting to the dentist entered simultaneously

**Variable**	**Odds Ratio**^1^	**95% CI**		***p *value**
		Lower	Upper	
*Age group*				
60+ years^2^	1.00			0.0002
18–39 years	4.14	2.12	8.07	0.0001
40–59 years	3.01	1.51	5.99	0.0017
				
*Sex*				
Male^2^	1.00			
Female	2.09	1.35	3.24	0.0010
				
*Social class*				
Non-manual^2^	1.00			
Manual	1.80	1.16	2.80	0.0088
				
*Education*				
University qualification^2^	1.00			
No university qualification	1.77	1.06	2.96	0.0288
				
*Self-reported visiting to the dentist*				
Regular^2^	1.00			
Irregular	2.26	1.48	3.46	0.0002

^1 ^Adjusted for the other variables in the table

^2 ^Reference category

## Discussion

These data support the performance of the MDAS as a measure of dental anxiety. The internal consistency was very high and the items appeared to describe a uni-dimensional construct which we would understand as providing a dimension of dental anxiety ranging from low to high. More sophisticated methods are available [25] to tease out discrepancies to this measurement model, however for practical purposes the user will find that the responses they receive from individual participants are easily interpretable.

This is the first report providing normative data of a representative sample of the UK general population. The level of high dental anxiety in the sample was 11% and is comparable to other reports from local or regional community surveys [6,11]. A large representative UK survey (N = 1800) using Corah's Dental Anxiety Scale has been reported which showed that 11% of their sample showed high dental anxiety (≥15) [26]. Caution should be taken when comparing studies that use different measures and cut-off values. For example, two 'conventional' cut-off values (≥13 and ≥15) are often used for data collected from Corah's scale (see for examples [27] and [26]). The cut-off of 19 was selected for the MDAS previously on empirical grounds, and provides greater confidence in the interpretation of the proportion that score at or above this point.

These figures are also reflected in the overall scores presented in the form of percentiles. The percentile at which men reach the 19 cut-off point was 90% in younger men (18 – 39 years) whereas in women this point was reached at the 85th percentile. However, an interesting interaction of gender with age was apparent, reflected in the much higher percentile (97th) in the oldest male age group (60+ years) compared with female counterparts (90th). A male patient aged 60+ years who scored 19 would be part of just 3% of the general population with the same or higher score whereas the comparable female would be associated with as many as 10% of the public. Moreover, a 25 year old male with a score of 9 could be informed by the dentist that about half of the people his age score lower than him, and by implication, half score higher.

The total scores for the MDAS varied by sex and age group but unlike the higher category of dental anxiety (e.g. extremely anxious or above the cut-off value), the interaction of age and sex was not significant. That is, it would appear that at more extreme levels of dental anxiety the effect of age and gender interacted so that in older age groups women were more dentally anxious than men and that these differences were stronger than at the lowest age group. The comparison of results between averages and proportions scoring at a clinically relevant cut-off across the factors of age and gender, was interesting and alerts the researcher and clinician to focus on the purpose and use of scores obtained from an instrument such as the MDAS. Caution is required when considering patients in higher scoring groups as previously mentioned. The validity of the cut-off, although empirically determined, requires additional support. The relatively small error contained in the measure should not be ignored and should indicate to the practitioner that repeated assessment at a later date would be prudent. A discussion with patients about their feelings associated with dental visiting would also assist in the assessment process, especially with those with high scores.

The population norms produced by this survey for the MDAS have merit as they are recently produced and are likely to be more representative. The original norms published in 1995 comprised of four groups of participants obtained in the first 2 years of that decade. They included industrial dental service and general practice service users, members of the community visiting their general medical practitioner (GMP) and mothers attending the community dental service with their child. The closest comparative group was the GMP visitors as they will comprise of participants who do not visit the dentist – a feature not apparent in the other 3 original groups. The mean (SD) levels of the current study (n = 963) and the 1995 'community' group (n = 525) were 10.36 (5.36) and 10.39 (5.46) respectively. These showed remarkable similarity (t = 0.10, df = 1486, *p *= .54). Another interesting comparison was to inspect the percentages of participants who rated their anxiety as 'extremely anxious'. These data were available for both the total (genders combined) samples in the original (page 146, table four) and current surveys. The level of the rating was identical for the anticipatory items (1 and 2) however the percentage who rated the injection as extremely anxious was 14.6% in 1993 and only 9.5% in 2008. The mean dental anxiety scores for those aged 60 and above were higher in males in the current survey compared to the original community sample (page 147; table five, males: mean = 7.52 (3.81) n = 122 *versus *6.52 (2.34) n = 50; t = 2.17, df = 170, *p *= .016), whereas females showed a smaller increase in the recent survey compared to the original, and the difference was not significant: 10.09 (5.57) n = 122 *versus *9.23 (5.25) n = 71; t = 1.07, df = 191, *p *= .146). There is partial support therefore for Locker's suggestion [28] that the 50–59 year old cohort 'will carry their relatively high levels of dental anxiety into old age' [29]. This effect appears to be shown in males only. These comparisons are tentative at best however, and await more extensive and organised data collection from longitudinal studies.

The major demographic variables were found to relate strongly with dental anxiety as shown in the multiple logistic regression analysis. Dental anxiety has been reported frequently in previous studies to vary with sex, age, education and social class. Of interest in particular for this study was the relationship of age group with the categorisation of high *versus *low/moderate dental anxiety. The benefit of conducting the multiple logistic regression was that the effects of education and social class were removed to allow a focus on the relationship of dental anxiety by age. No evidence could be found for a reduction of the proportion of participants with high dental anxiety in the youngest age group. Rather the likelihood of being highly dental anxious compared to those 60 years of age or more was four times greater. This finding supports the view that dental anxiety is relatively stable as a construct regardless of changes to treatment delivery. An interesting possibility may be that to achieve reductions in dental anxiety dental staff may need to engage more with their patients, probably at the young end of the age spectrum to develop resilience. The employment of active and sensitive communication skills may enhance this process [30].

Potential limitations of this study should be considered. Every effort was made by the market research company to ensure a representative sample. The original spreadsheets of the age, gender and social status breakdown showed that the discrepancy between what the survey aimed to collect to attain representativeness and what was actually collected was found to be similar. Admittedly there was a minor over-representation of ABC respondents. Residential location however was a good fit. The strength of association between social class and dental anxiety was one of the weakest factors entered into the logistic regression (together with education). Hence a weighting procedure to the analysis was not considered to be warranted due to the increased complexity of introducing such a procedure. The response rate of 14% was admittedly low although as argued immediately above we believe the potential bias was minimal. Other authors using telephone survey techniques also experienced similar problems (with an 18.5% response rate) and use identical arguments to our own to support the veracity of the data set [28]. However, a risk of sampling bias exists with telephone survey methods if response rates vary in different groups within a population. For example, some may not have a landline telephone and may only use mobile telephones. Although the impact of such potential bias is unknown, other methods are also prone to response bias for similar reasons. For example, postal surveys can lead to over representation of the views of white participants with higher incomes and educational attainment [31]. Telephone interviews have been used in national dental surveys [32] and remain an important method for public health and social surveys seeking populations' views, particularly in North America [33-35].

## Conclusion

This new dataset has determined that the MDAS showed high reliability and excellent completion of scale items. No support was found for reduced dental anxiety in younger age groups. The set of norms produced will be useful for dentists and researchers when interpreting individuals' expression of dental anxiety through the MDAS questionnaire.

## Competing interests

The authors declare that they have no competing interests.

## Authors' contributions

GH, TD and PR conceived and designed the study. GH analysed the data, drafted the article and subsequent versions. TD and PR coordinated the data collection via the survey organization, contributed to the manuscript, and edited all drafts. All authors read and approved the final manuscript.

## Pre-publication history

The pre-publication history for this paper can be accessed here:


